# Cooperative Jahn–Teller effect and the role of strain in the tetragonal-to-cubic phase transition in Mg*_x_*Cu_1 − *x*_Cr_2_O_4_

**DOI:** 10.1107/S2052252516012574

**Published:** 2016-08-16

**Authors:** Serena C. Tarantino, Mattia Giannini, Michael A. Carpenter, Michele Zema

**Affiliations:** a Department of Earth and Environmental Sciences, University of Pavia, Via Ferrata 9, 27100 Pavia, Italy; b CNR, Institute of Geosciences and Georesources, Via Ferrata 9, 27100 Pavia, Italy; c Department of Earth Sciences, University of Cambridge, Downing Street, Cambridge, Cambridgeshire CB2 3EQ, UK

**Keywords:** spinel, X-ray diffraction, Jahn–Teller distortion, phase transition

## Abstract

Temperature and composition dependences of the *I*4_1_/*amd* →  phase transition in the Mg*_x_*Cu_1 − *x*_Cr_2_O_4_ spinel solid solution, due to the melting of the cooperative Jahn–Teller distortion, have been studied by means of single-crystal X-ray diffraction. Crystals with *x* = 0, 0.10, 0.18, 0.43, 0.46, 0.53, 1 were grown by flux decomposition methods. All crystals have been refined in the tetragonal *I*4_1_/*amd* space group except for the Mg end-member, which has cubic symmetry. In Mg*_x_*Cu_1 − *x*_Cr_2_O_4_ the progressive substitution of the Jahn–Teller, *d* ^9^ Cu^2+^ cation with spherical and closed-shell Mg^2+^ has a substantial effect on the crystal structure, such that there is a gradual reduction of the splitting of *a* and *c* unit-cell parameters and flattening of the tetrahedra. Single-crystal diffraction data collected *in situ* up to *T* = 1173 K show that the tetragonal-to-cubic transition temperature decreases with increasing Mg content. The strength of the Cu—Cu interaction is, in effect, modulated by varying the Cu/Mg ratio. Structure refinements of diffraction data collected at different temperatures reveal that heating results in a gradual reduction in the tetrahedron compression, which remains significant until near the transition temperature, however, at which point the distortion of the tetrahedra rapidly vanishes. The spontaneous strain arising in the tetragonal phase is large, amounting to 10% shear strain, *e*_t_, and ∼ 1% volume strain, *V*_s_, in the copper chromite end-member at room temperature. Observed strain relationships are consistent with pseudoproper ferroelastic behaviour ( ∝ *V*_s_ ∝ , where *q*_JT_ is the order parameter). The *I*4_1_/*amd* →  phase transition is first order in character for Cu-rich samples and then evolves towards second-order character. Although a third order term is permitted by symmetry in the Landau expansion, this behaviour appears to be more accurately represented by a 246 expansion with a change from negative to positive values of the fourth-order coefficient with progressive dilution of the Jahn–Teller cation.

## Abstract 2

In the Mg*_x_*Cu_1 − *x*_Cr_2_O_4_ solid solution, progressive substitution of the Jahn–Teller and *d* ^9^ Cu^2+^ cation with the spherical and closed-shell Mg^2+^ cation results in a gradual reduction of the splitting of *a* and *c* unit-cell parameters, until transformation occurs from the tetragonal *I*4_1_/*amd* to the cubic 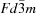 archetype spinel structure. Tetragonal members of the series transform to cubic at high temperature, and the transition temperature decreases with increasing Mg content. The phase transition is first order in character for Cu-rich samples and evolves towards second-order character at intermediate compositions.

## 1. Introduction   

Complex *AB*_2_O_4_ oxides with the spinel structure comprise a family of materials, which exhibit a wide range of electronic, magnetic and optical properties through the variation of cations on tetrahedral (*A*) and octahedral (*B*) sites. The archetypical spinel structure belongs to the space group 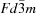 (No. 227) and is usually described as a pseudocubic close-packed array of O atoms with the *A* and *B* cations occupying one eighth of the tetrahedral sites and one half of the octahedral sites, respectively. Such occupancy of the interstitial sites results in an *fcc* unit cell which is 2 × 2 × 2 times that of the basic *ccp* oxygen array. One of the characteristics of the spinel structure is its flexibility in the range of possible cations and cation charge combinations, making it a structure adopted by over a hundred compounds. In fact, within the spinel space group, the fractional coordinates of the octahedral and tetrahedral sites are fixed at special positions (*A* on 8*a* at 0,0,0; *B* on 16*d* at 5/8,5/8,5/8), while the O atoms are on 32*e* with coordinates *uuu*. This means that if the relative sizes of the *A* and *B* cations change, their positions remain the same but the oxygen array expands or contracts to accommodate them and maintain the same symmetry throughout.

**Figure 1:**
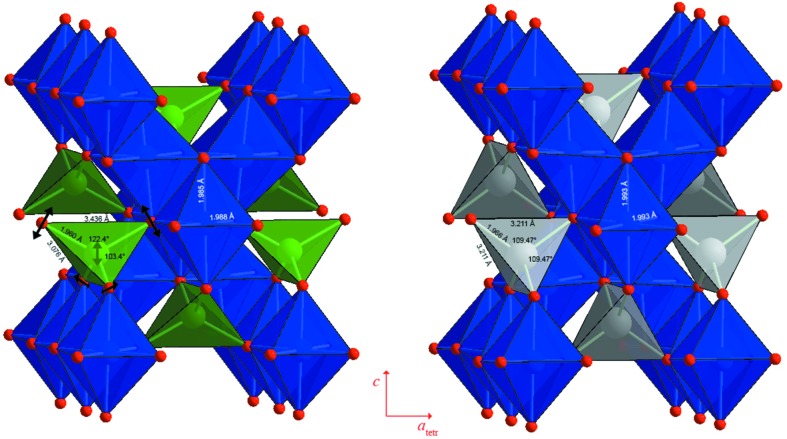
Perspective views of the crystal structures of CuCr_2_O_4_ (left) and MgCr_2_O_4_ (right) along the *a*-axis of the *I*4_1_/*amd* cell. CuO_4_ tetrahedra are drawn in green, MgO_4_ tetrahedra in grey. In CuCr_2_O_4_, the contraction along the *c* direction due to JT flattening of tetrahedral sites is given by a change of tetrahedral angles. Relevant geometrical parameters are reported.

The most common distortion of the spinel structure is by far the tetragonal distortion, whereby one of the cubic axes would become compressed or elongated with respect to the other two. If no additional symmetry breaking occurs, the tetragonal distortion alone decreases the symmetry from 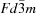 to *I*4_1_/*amd* (No. 141). The *c*/*a* ratio is normally used as a parameter of tetragonal distortion. A phase transition from the cubic to the tetragonal structure may be induced by a sufficient concentration of non-spherical, Jahn–Teller (JT) ions, such as Cu^2+^ or Mn^3+^, causing a cooperative distortion. Although less common, tetrahedral Cu^2+^ on the *A* site can display JT activity. The degeneracy of the partially occupied *t*_2_ levels is broken by compressing the tetrahedron and thereby lowering the symmetry, as in copper chromite, CuCr_2_O_4_, which is a tetragonally distorted spinel with unit-cell parameters ratio *c*/*a* < 1 (Fig. 1 ▸, left panel). Cu^2+^ cations can be stabilized in flattened tetrahedral environments because of the preference of Cr^3+^ ions to occupy the octahedral sites. The cooperative nature of the crystal distortion can be rationalized in terms of elastic interactions among locally distorted tetrahedra, as a consequence of coupling of electronic states to bulk deformation *via* elastic strain. On heating, CuCr_2_O_4_ undergoes a first-order structural transition from the tetragonal distorted spinel structure to the archetypal cubic spinel structure at 853 K (Yé *et al.*, 1994 ▸; Kennedy & Zhou, 2008 ▸). The structural distortion in CuCr_2_O_4_ is large and the transition temperature high, in particular if considering that CuO_4_ tetrahedra are not directly linked but separated from each other by non-JT ions. Nonetheless, enhancement of the ground-state JT splitting and of lattice distortion have been explained by considering the electronic and elastic coupling of Cu^2+^ and Cr^3+^ (Atanasov *et al.*, 1993 ▸; Reinen *et al.*, 1988 ▸). The relevance of strain associated with the Jahn–Teller distortion is curiously showed in NiCr_2_O_4_ by the observation that the crystals literally jump off a flat surface when they pass through the transition point (Crottaz *et al.*, 1997 ▸), due to the large and abrupt change in shear strain.

MgCr_2_O_4_ forms in the cubic spinel structure (Fig. 1 ▸, right panel). At room temperature (RT), the Mg-rich (*x* > 0.6) members of the Mg*_x_*Cu_1 − *x*_Cr_2_O_4_ solid solution are cubic, whereas the Cu-rich members (*x* < 0.43) are tetragonal. A two-phase region separates the cubic and tetragonal phases (Shoemaker & Seshadri, 2010 ▸; De *et al.*, 1983 ▸).

In this work, the effects on the crystal structure and on the tetragonal-to-cubic phase transition of progressive substitution of the Jahn–Teller and *d* ^9^ Cu^2+^ cation with the spherical and closed-shell Mg^2+^ cation in the Mg*_x_*Cu_1 − *x*_Cr_2_O_4_ solid solution have been studied. Given the relevance of strain in determining the structure-electronic properties relation, *in situ* high-temperature (HT) single-crystal diffraction data are analysed in terms of the evolution of symmetry-adapted strains for samples with different compositions along the joint Mg*_x_*Cu_1 − *x*_Cr_2_O_4_. Observed variations of spontaneous strains accompanying phase transitions are expected to provide detailed insights into the transition mechanisms.

## 2. Experimental   

### 2.1. Synthesis and crystal growth   

Single crystals of cubic Mg-rich and tetragonal Cu-rich chromites belonging to the series Mg*_x_*Cu_1 − *x*_Cr_2_O_4_ were grown by flux decomposition methods. The synthesis of single crystals of the Mg end-member was conducted on the basis of the strategy reported by Lenaz *et al.* (2004 ▸). Starting compounds were CuO (Fluka, > 99%), MgO (Carlo Erba, > 99%) and Cr_2_O_3_ (Merck, 99%). Na_2_B_4_O_7_ was used as a flux in a weight ratio of 2.2 with respect to the reactive mixture. The mixture was submitted to the following heating cycle: (1) heating from RT to 1473 K at a rate of 100 K h^−1^; (2) soaking at 1473 K for 24 h; (3) cooling to 1173 K at 6 K h^−1^; (4) isothermal heating at 1173 K for 9 h; (5) rapid cooling to RT. The residue was then washed with warm HCl 20%.

For the syntheses of the tetragonal Cu-rich members of the Mg*_x_*Cu_1 − *x*_Cr_2_O_4_ solid solution, the method reported by Yé *et al.* (1994 ▸) for growing crystals of CuCr_2_O_4_ has been adapted to different Cu/Mg stoichiometric ratios. Starting compounds were CuO (Fluka, > 99%), MgO (Carlo Erba, > 99%) and K_2_Cr_2_O_7_ (Carlo Erba, > 99%). K_2_Cr_2_O_7_ transforms into Cr_2_O_3_, which, when freshly formed, is highly reactive towards copper oxide. Potassium dichromate acts as a reactive flux for the crystal growth and an excess amount was therefore added according to
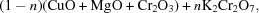
where *n* was chosen to be equal to 0.2 mol. B_2_O_3_ (1 wt%) was added to the mixture in order to increase the homogeneity of the solution and hence improve the quality of the crystals. Different CuO/MgO stoichiometric ratios were used to obtain spinels with nominal compositions: CuCr_2_O_4_ (*x* = 0), Mg_0.05_Cu_0.95_Cr_2_O_4_ (*x* = 0.05), Mg_0.1_Cu_0.9_Cr_2_O_4_ (*x* = 0.1), Mg_0.4_Cu_0.6_Cr_2_O_4_ (*x* = 0.4). The mixtures were submitted to the following heating cycle: (1) heating from RT to 1093 K at 100 K h^−1^; (2) soaking at 1093 K for 24 h; (3) cooling at 30 K h^−1^. Given the high refractory properties of MgO, stage (2) was prolonged for 115 h in the case of the mixture with *x* = 0.4. After the thermal runs, the residues were washed with boiling water and the single crystals removed from the solidified flux.

### 2.2. Single-crystal XRD at room temperature   

Several crystals were isolated from each synthesis residue. They were checked for crystal quality by analysing X-ray diffraction profiles. The selected crystals, labelled Cu100, Cu90, Cu82, Cu57, Cu47 and Mg100 on the basis of their actual compositions as determined from structure refinements and electron-microprobe analyses (see §§2.4 and 2.5), were submitted to single-crystal diffraction analysis at RT using a Bruker-AXS APEX diffractometer equipped with a CCD detector. Data collections were carried out with operating conditions 50 kV and 30 mA and graphite-monochromated Mo *K*α radiation (λ = 0.7107 Å). The Bruker SMART system of programs was used for preliminary crystal lattice determination and X-ray data collection. A total of 4800 frames (resolution: 512 × 512 pixels) were collected with eight different goniometer settings using the ω-scan mode (scan width: 0.3°ω; exposure time: 5–20 s per frame, depending on the size and relative scattering power of the crystals analysed; detector–sample distance: 60 mm). Complete data collection was achieved up to sin θ/λ *ca* 0.95 Å^−1^.

All the tetragonal crystals of the series were twinned, as expected given the synthesis conditions which imply the use of high temperature, where the cubic phase is stable, and subsequent transformation to tetragonal on cooling. *I*4_1_/*amd* is a maximal nonisomorphic *t*-subgroup of 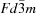 and the formation of ferroelastic domains, including transformation twinning, is inevitable during the phase transition. The corresponding ferroelastic species according to Aizu’s notation (Aizu, 1969 ▸) is 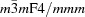, where ‘F’ stands for ferroic, and separates the parent point group (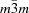) from the derived point group (4/*mmm*). As 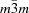 and 4/*mmm* are of the order 48 and 16, respectively, there are three possible orientation states in the tetragonal phase. In our study, three twin components were found to be present in crystal Cu100, while two components were detected in the other tetragonal crystals.

The Bruker program *SAINT*+ was used for the data reduction, including intensity integration, background and Lorentz–polarization corrections. Intensity data from the twin components present in tetragonal crystals were integrated taking into account the superposition affecting some diffraction spots. Final unit-cell parameters were obtained by the Bruker *GLOBAL* least-squares orientation matrix refinement procedure, based on the positions of all measured reflections. The semi-empirical absorption correction of Blessing (1995 ▸), based on the determination of transmission factors for equivalent reflections, was applied using the Bruker programs *SADABS* or, for twinned crystals, *TWINABS* (Sheldrick, 2003 ▸). Details of room-temperature data collection by the CCD diffractometer are reported in Table 1 ▸.

### 2.3. Single-crystal XRD at high temperature   

Crystals Cu100, Cu90, Cu82, Cu54 (not measured at RT by the CCD diffractometer) and Mg100 were submitted to *in situ* high-temperature single-crystal diffraction investigations using a Philips PW1100 four-circle diffractometer with point-counter detector. Crystals Cu57 and Cu47 were not used for the HT study due to their low diffracted intensities. Operating conditions were 55 kV and 30 mA with graphite-monochromated Mo *K*α radiation (λ = 0.7107 Å). Horizontal and vertical apertures of the point counter detector were 2.0 and 1.5°, respectively. High-temperature measurements were performed by using a home-made U-shaped microfurnace, which has been in use in our laboratory for over 15 years. It makes use of a Pt–Pt/Rh resistance, which allows temperatures up to 1273 K to be achieved, and is equipped with a K-type thermocouple. Temperature calibration (calibration curve *R* ^2^ = 0.9994) is regularly done by known melting points of several pure compounds and by the transition temperature of quartz (Carpenter, Salje, Graeme-Barber, Wruck *et al.*, 1998 ▸). Reported temperatures are precise to within ±5 K in the whole temperature range. The design of the furnace limits the angular excursion of the ω circle to *ca* 27.5° (sin θ/λ *ca* 0.65 Å^−1^ with Mo *K*α radiation). As routinely done for HT measurements using this system, the selected crystals were inserted into quartz capillaries (0.3–0.5 mm Ø, depending on the dimensions of the crystals) and kept in position by means of quartz wool in order to avoid any mechanical stress. Unit-cell parameters were measured from RT up to 1173 K at regular steps. At each working temperature, the orientation matrix was updated by centring 24 reflections selected in the range of sin θ/λ *ca* 0.2–0.34 Å^−1^, and accurate lattice param­eters (reported in Tables 2 ▸ and 3 ▸ for tetragonal and cubic phases, respectively) were derived from a least-squares procedure based on the Philips LAT routine over up to 60 d*-spacings, each measured from the positions of all reflection pairs at ±θ in the range of sin θ/λ 0.073–0.628 Å^−1^.

For each crystal, complete datasets of diffracted intensities were collected at different temperatures, both below and above the phase transition temperature, using the same operating conditions as reported above. Intensity data were measured in the sin θ/λ range 0.05–0.628 Å^−1^ in the ω-scan mode (2.0° θ scan width; 0.05° θ s^−1^ scan speed). Only diffraction spots belonging to the orientation matrix of the main twin component were measured, thus including overlapping reflections. Three standard reflections were collected every 200 measured reflections. X-ray diffraction intensities were obtained by measuring step-scan profiles and analysing them by the Lehmann & Larsen (1974 ▸) σ*_I_*/*I* method, as modified by Blessing *et al.* (1974 ▸). Intensities were corrected for absorption using the semi-empirical φ-scan method of North *et al.* (1968 ▸). Relevant parameters for data collected at different temperatures are reported in Table 4 ▸. Some reflections, representative of different classes, were also scanned periodically (ω/2θ scan mode; 2.0° θ scan width; 0.1° θ s^−1^ scan speed) to check for the crystallinity of the sample.

### 2.4. Structure refinements   

All structure refinements were carried out by full-matrix least-squares using *SHELXL*97 (Sheldrick, 2008 ▸). Equivalent reflections were averaged, and the resulting internal agreement factors *R*_int_ are reported in Table 1 ▸ for all the datasets collected at RT, and in Table 4 ▸ for datasets collected at HT. The atomic scattering curves were taken from *International Tables for X-ray Crystallography* (Ibers & Hamilton, 1974 ▸). For datasets collected at RT by the CCD diffractometer, contributions from the different twin components were taken into account by using the HKLF-5 format in *SHELXL*97 and including the BASF parameter in the refinement. For all structure refinements, structure factors were weighted according to *w* = 1/[σ^2^(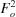) + (*AP*)^2^ + *BP*], where 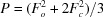, and *A* and *B* were chosen for every crystal to produce a flat analysis of variance in terms of 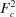, as suggested by the program. An extinction parameter *x* was refined to correct the structure factors according to the equation: 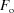 = 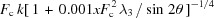 (where *k* is the overall scale factor). In addition to *x* and *k*, atomic positions, anisotropic displacement parameters and site occupancy at the *A* site (for terms with intermediate composition) were refined simultaneously. The Cu/Mg ratios obtained from unconstrained refinements of site occupancy were close to the nominal compositions and were then confirmed by electron microprobe analyses performed on the same crystals at the end of the HT experiments (see §2.5). Final difference-Fourier maps were featureless. Values of the conventional agreement indices, *R*_1_ and *R*_all_, as well as the goodness of fit (S) are reported in Tables 1 ▸ and 4 ▸ for RT and HT datasets, respectively, whereas interatomic distances and selected geometrical parameters are reported in Table 5 ▸ for the RT datasets and in Tables 6–10 ▸ ▸ ▸ ▸ ▸ for the HT datasets. Atomic fractional coordinates, anisotropic displacement parameters *U*_*ij*_ and lists of observed and calculated structure factors are available in the CIF files of supporting information.

### 2.5. Electron probe microanalyses (EPMA)   

At the end of the diffraction experiments, all the crystals used in the present study were embedded in epoxy resin, polished and analysed by electron microprobe. The chemical compositions were measured with a Jeol JXA-8200 electron microprobe, fully automated with 5 crystals and 5 wavelength dispersive spectrometers. The polished samples were coated with about 10 nm of amorphous carbon to avoid charging of the surface and studied at acceleration voltages of 15 kV and probe current of 15 nA. The analytical standards used for the calibration of the energy position of the analyzed elements were Cu_2_O, MgO and Cr_2_O_3_ for Cu, Mg and Cr, respectively. For each sample, 10 to 15 points were measured and the averaged chemical compositions, as well as the corresponding standard deviations, are reported in Table 5 ▸.

## 3. Results and discussion   

### 3.1. Unit-cell parameters and geometry of tetrahedra at RT   

**Figure 2:**
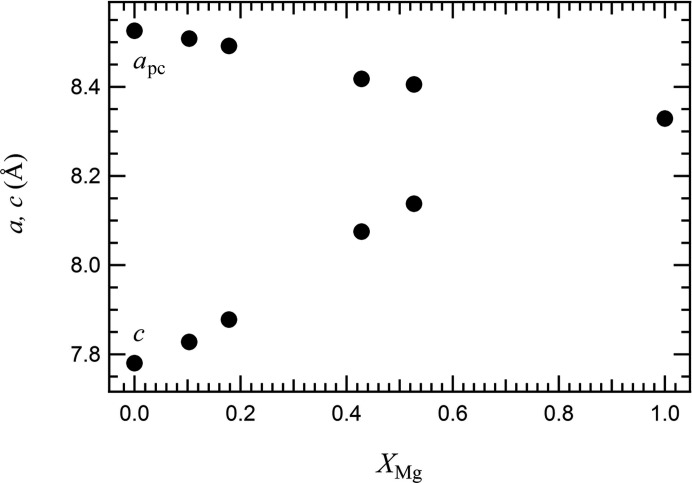
Variation of unit-cell parameters as a function of composition at room temperature across the Mg*_x_*Cu_1 − *x*_Cr_2_O_4_ join. For ease of comparison, the *a* parameter in the low-temperature tetragonal phase has been scaled and displayed in the pseudocubic setting 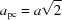. The vertical size of the symbols exceeds the uncertainties in unit-cell parameters.

Refinements of X-ray diffraction data reveal a high sensitivity of the crystal structure to the amount of Cu^2+^ present. The unit-cell parameters are reported as a function of Mg content in Fig. 2 ▸. They are expressed in terms of the cubic unit cell itself (MgCr_2_O_4_) or of the reduced pseudocubic cell (*I*4_1_/*amd* structures of the Cu-rich samples). Samples with *x* ≤ 0.53 are isostructural with CuCr_2_O_4_, thus the tetragonal region seems slightly larger than previously reported by De *et al.* (1983 ▸), in agreement with recent data of Shoemaker & Seshadri (2010 ▸). Across the solid solution, starting from tetragonal CuCr_2_O_4_, replacement of Cu by Mg is accompanied by an increase in the *c*-axis and a decrease in the *a*-axis lengths, and hence leads to a gradual decrease of the tetragonal distortion.

**Figure 3:**
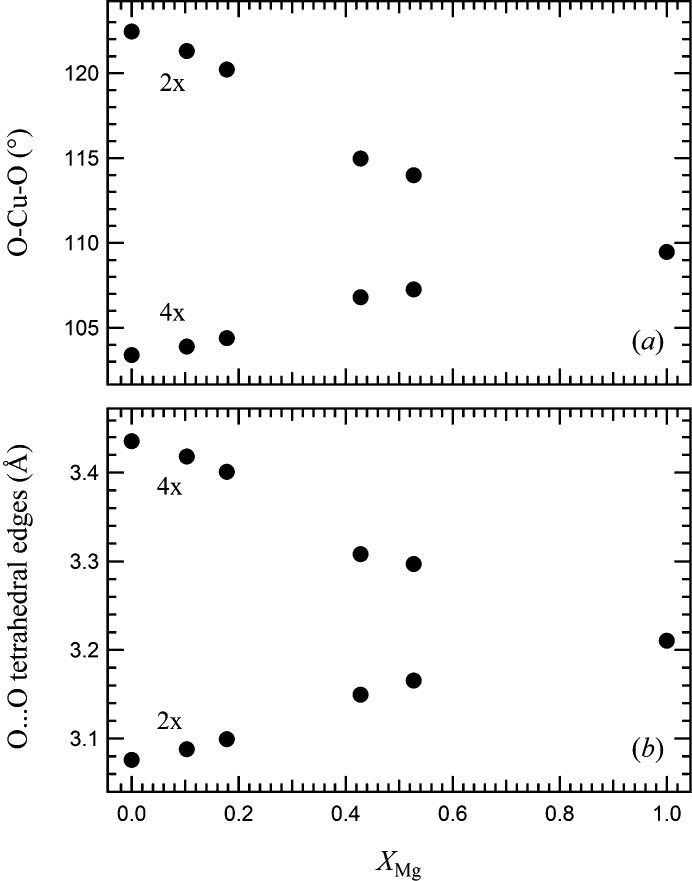
Variation of (*a*) tetrahedral O—(Cu,Mg)—O angles and (*b*) O⋯O edges as a function of Mg content. Error bars are within the symbols.

The influence of the Jahn–Teller effect can be better estimated by looking at the geometry of the tetrahedra. These are flattened and, with respect to the ideal tetrahedron, display four smaller and two angles larger than 109.47° (see Fig. 1 ▸ for visual reference). The distortion of the tetrahedra is large, with ΔO—Cu—O = 12.94° in the Cu end-member. The distortion of the tetrahedra in the tetragonal phase is also evident in the behaviour of the O⋯O edges. In Fig. 3 ▸, O—(Cu,Mg)—O angles and O⋯O edges are plotted as a function of composition. With increasing Mg content, the flattening of the tetrahedra is reduced: the two sets of O—(Cu,Mg)—O tetrahedral angles converge towards 109.47°, as required by the 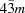 site symmetry of the cubic phase. A similar behaviour is shown by the tetrahedral edges. Variation of the (Cu,Mg)—O bond length (Table 5 ▸) is related to the difference in ion size between Cu^2+^ and Mg^2+^.

**Figure 4:**
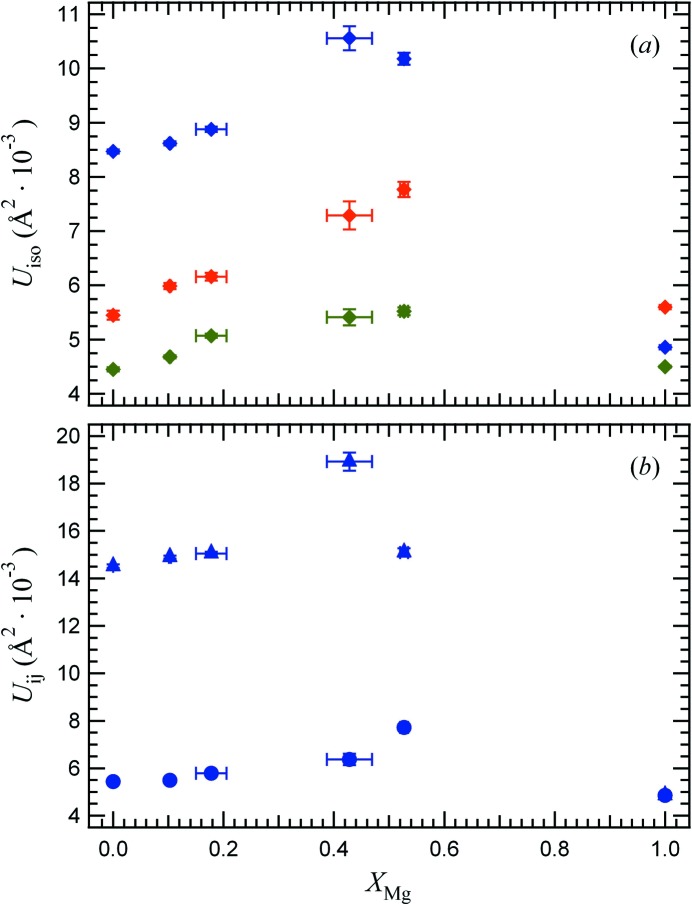
Variation of atomic displacement parameters as a function of composition: (*a*) isotropic ADPs of Cu/Mg (blue diamonds), Cr (green diamonds) and O (red diamonds); (*b*) anisotropic APDs *U*_11_ (circles) and *U*_33_ (triangles) for the Cu/Mg site.

Homogeneity of the solid solution is quite good. EPMA spot analyses reveal a rather narrow composition range within each sample (Table 5 ▸), with Cu54 and Cu47 showing the highest e.s.d.s. When looking at the equivalent atomic displacement parameters (ADPs; Fig. 4 ▸ *a*), crystals with intermediate compositions show slightly higher values than those of the two end-members due to some static disorder, with an overall behaviour that is common for solid solutions. Interestingly and as already reported previously (*e.g.* Kennedy & Zhou, 2008 ▸), in all Cu-bearing crystals, the Cu/Mg site is the one showing the highest displacement parameters. This is mainly due to an elongated displacement ellipsoid towards the *c*-axis (Fig. 4 ▸ *b*). The *R*_max_/*R*_min_ ratio of the principal axes of the thermal ellipsoid is 2.67 for the tetrahedral cation in Cu100 and decreases almost linearly with increasing Mg content, with Cu47 slightly deviating from this trend likely due to some compositional heterogeneity. However, the behaviour observed for the average structure by XRD does not necessarily allow to differentiate the distinct cation coordinations of Mg^2+^ and Cu^2+^ if they are different on the local length scale.

### 3.2. High-temperature behaviour   

**Figure 5:**
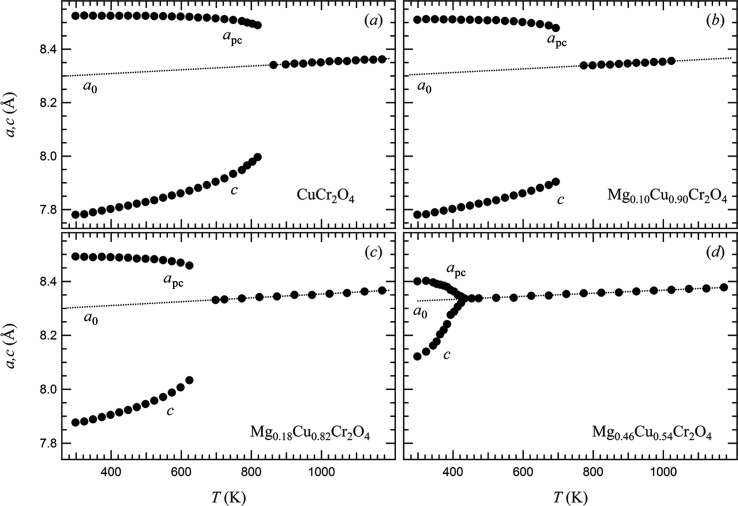
Variation as a function of temperature of unit-cell parameters for spinel samples of different compositions across the Mg*_x_*Cu_1 − *x*_Cr_2_O_4_ join. (*a*) CuCr_2_O_4_; (*b*) Mg_0.10_Cu_0.90_Cr_2_O_4_; (*c*) Mg_0.18_Cu_0.82_Cr_2_O_4_; (*d*) Mg_0.46_Cu_0.54_Cr_2_O_4_. The values of *a*_0_ are the lattice parameters of the respective cubic parent phase extrapolated from high temperature into the stability field of the tetragonal phase.

The temperature dependence of the lattice parameters for CuCr_2_O_4_ and all the intermediate compounds of the series Mg*_x_*Cu_1 − *x*_Cr_2_O_4_ is shown in Fig. 5 ▸. Heating the samples results in a gradual reduction of the splitting of *a* and *c* unit-cell parameters. Variation of the unit-cell parameters with temperature for CuCr_2_O_4_ is in good agreement with previously reported data (Kennedy & Zhou, 2008 ▸) and shows (Fig. 5 ▸ *a*) a large first-order jump above 818 K, when the tetragonal splitting is abruptly lost. By inspection of the variations of the lattice parameters for the samples of intermediate compositions (Figs. 5 ▸ *b*–*d*), it is possible to note how the gradual substitution of Cu for Mg causes a reduction of the initial *a*–*c* splitting and of the discontinuity at the transition, and a shift of the transition temperature towards lower temperatures.

The evolution of lattice parameters of cubic phases have been fitted with straight lines, yielding the following thermal expansion α*_a_* coefficients: Cu100: 7.1 (3) × 10^−5^ K^−1^; Cu90: 6.9 (3) × 10^−5^ K^−1^; Cu82: 7.1 (3) × 10^−5^ K^−1^; Cu54: 5.9 (2) × 10^−5^ K^−1^. The reported values are all very similar and in good agreement with values reported for most cubic spinels. Variations of the cubic reference parameters, *a*_0_, for determination of spontaneous strains of the tetragonal phase were obtained by extrapolation to lower temperatures of these fits. The *a* and *c* parameters of the tetragonal phase do not converge symmetrically into the extrapolated values of *a*_0_ for the cubic phase because of a volume expansion associated with the transition.

**Figure 6:**
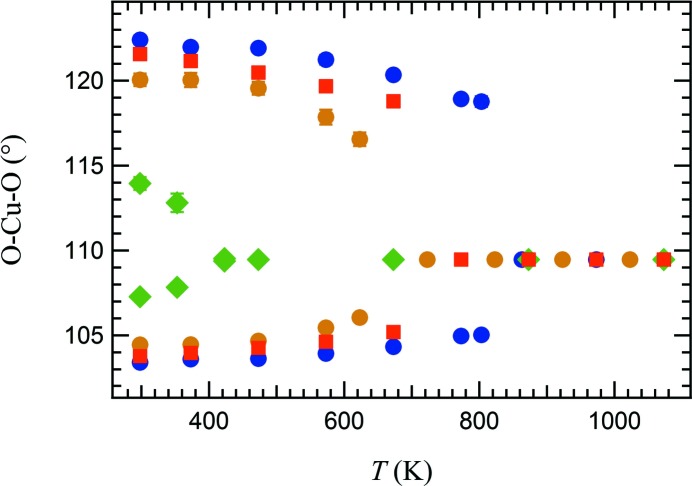
Variation of tetrahedral O—(Cu,Mg)—O angles as a function of temperature. Blue: CuCr_2_O_0_; red: Mg_0.10_Cu_0.90_Cr_2_O_4_; orange: Mg_0.18_Cu_0.82_Cr_2_O_4_; green: Mg_0.46_Cu_0.54_Cr_2_O_4_. Error bars are within the symbols.

The temperature dependence of the angles within the (Cu,Mg)O_4_ tetrahedron is illustrated in Fig. 6 ▸ for all the analysed Cu-rich samples. Heating results in a gradual reduction of the compression of the tetrahedron but, clearly, the compression remains significant until near the transition temperature. The variation with temperature of the O—(Cu,Mg)—O angles for the samples of intermediate composition mimics the change in the lattice parameters, the discontinuity of distortion at the transition decreases when Mg is added.

The nature of JT transitions, cooperative or order–disorder, at the solid state has been debated at length and both scenarios observed in several phases (for a review see, *e.g.*, Kugel & Khomskii, 1982 ▸; Goodenough, 1998 ▸; Bersuker, 2006 ▸). Similarly, in copper spinel, two mechanisms for the JT transition can be proposed: in the first case, upon increasing *T*, all tetrahedra transform from flattened in the JT distorted spinel to ideal in the cubic spinel; in the second case, the energy-lowering cation coordination distortions persist above the structural transition temperature and CuO_4_ tetrahedra are always locally JT distorted. In the latter hypothesis, the structural transition has to be regarded as an order–disorder transition, at which local JT distortions become spatially uncorrelated, although do not disappear; the average crystal structure, which would in turn result in regular tetrahedra, accounts for this disorder through increased thermal displacement parameters.

In the case of JT transitions in systems with octahedrally coordinated cations, as in the case of manganite perovskites, large ADPs for O atoms are observed in the disordered phase along the cation—O bonds, reflecting the largest static bond length distribution (*i.e.* a mixture of long and short bonds) in these directions. In CuCr_2_O_4_, the distortions involve the tetrahedral angles, ADPs of Cu and O atoms are not elongated in a direction parallel to Cu—O bonds. ADPs of O atoms are elongated perpendicularly to the Cu—O bonds, whereas those of Cu atoms are elongated along the *c*-axis. Both these apparent vibrations affect the tetrahedral angles rather than bond lengths and may indicate the presence of disorder at the Cu sites in the tetragonal phase.

**Figure 7:**
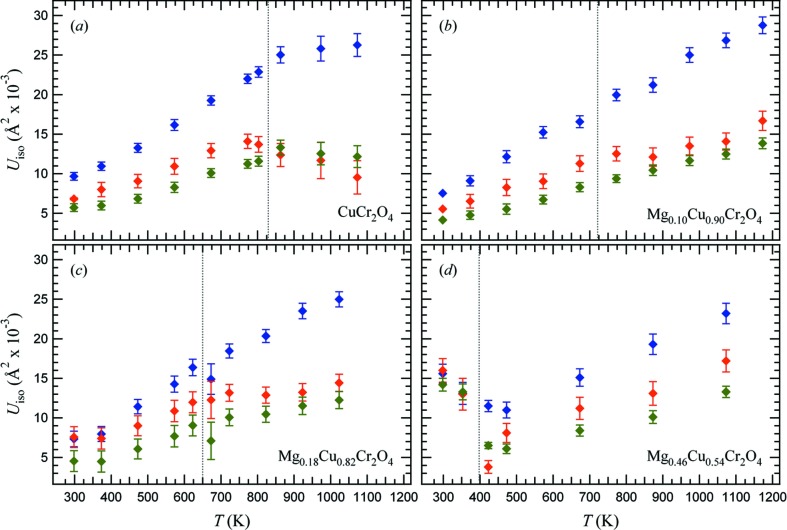
Variation as a function of temperature of isotropic atomic displacement parameters of Cu/Mg (blue diamonds), Cr (green diamonds) and O (red diamonds) atoms for spinel samples of different compositions across the Mg*_x_*Cu_1 − *x*_Cr_2_O_4_ join. (*a*) CuCr_2_O_4_; (*b*) Mg_0.10_Cu_0.90_Cr_2_O_4_; (*c*) Mg_0.18_Cu_0.82_Cr_2_O_4_; (*d*) Mg_0.46_Cu_0.54_Cr_2_O_4_.

The temperature dependence of the isotropic atomic displacement parameters for Cu, Cr and O atoms in CuCr_2_O_4_ and the intermediate compounds of the series Mg*_x_*Cu_1 − *x*_Cr_2_O_4_ is shown in Fig. 7 ▸. With increasing temperature up to the transition, the isotropic ADPs of the Cu-rich samples increase linearly, as expected within the harmonic approximation at *T* far from 0 K. All tetragonal datasets have linear extrapolation to 0 K that are negligible within 3σ. This indicates the absence of a significant static disorder effect. In samples Cu90 and Cu82, this may be interpreted by considering that the ionic radii of both Mg^2+^ and Cu^2+^ in coordination 4 is 0.57 Å (Shannon, 1976 ▸). On the other hand, large scatter is displayed at low *T* by the ADPs of Cu54 sample, which shows a less clear trend in the tetragonal phase, likely due to compositional heterogeneities.

For all samples, a discontinuity in the temperature evolution of ADPs can be observed at the transition temperature. The observed behaviour of ADPs would suggest the transition to be driven by cooperative distortion, although the presence of an order–disorder component cannot be excluded on the basis of average structure information only.

### 3.3. Spontaneous strain in CuCr_2_O_4_   

Instability of the electronic structure is the driving mechanism for a Jahn–Teller transition. However, the change in the structural state appears overtly as changes in lattice parameters, and these can be described formally in terms of macroscopic strains. Variations of spontaneous strains accompanying a phase transition can be used to quantify the associated order parameters and are expected to provide detailed insights into the mechanisms of the transition itself. Strain parameters have been calculated by using the equations given by Carpenter, Salje & Graeme-Barber (1998 ▸), who reviewed the use of spontaneous strain to measure order parameters associated with phase transitions in minerals. In this case, there are two strains, the symmetry-breaking tetragonal shear strain, 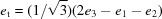, where 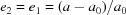, 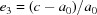, and the volume strain, 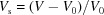.

**Figure 8:**
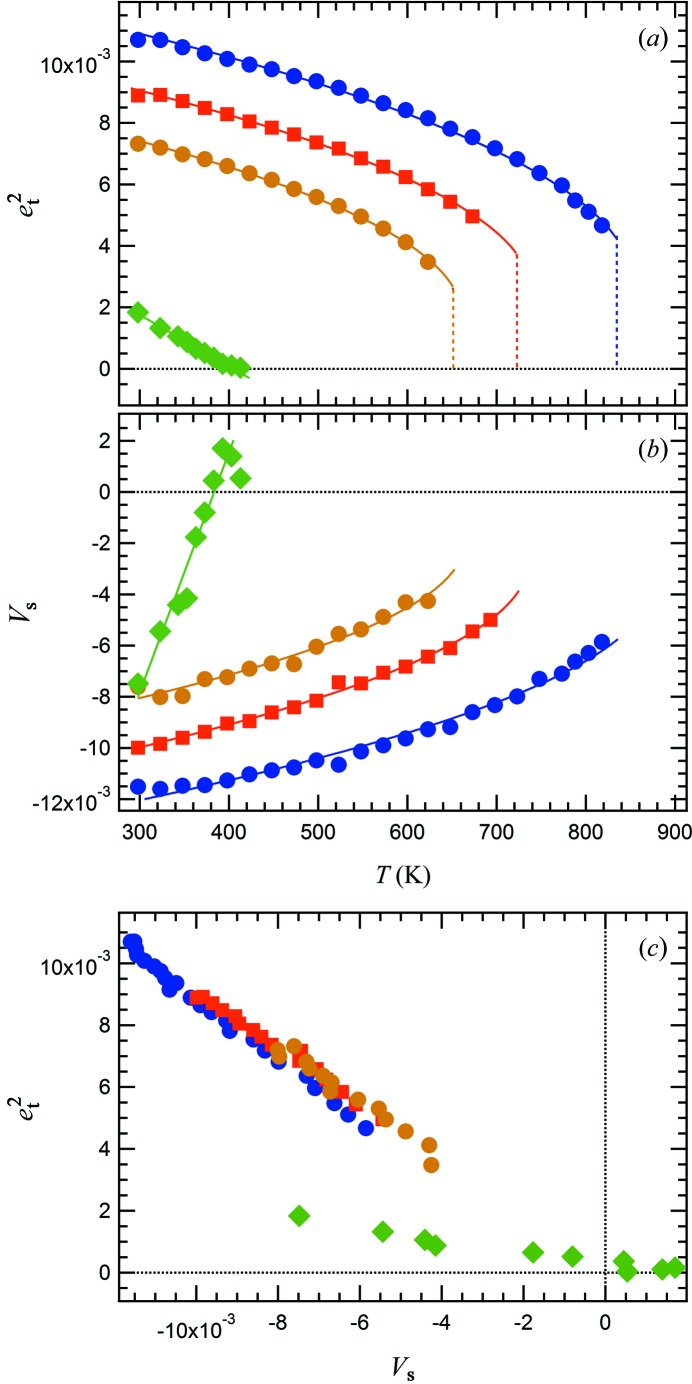
Symmetry-adapted strains calculated from lattice parameters for Mg*_x_*Cu_1 − *x*_Cr_2_O_4_ with *x* = 0, 0.10, 0.18, 0.46. (*a*) The symmetry-adapted tetragonal strains follow the classical pattern of a first-order phase transition driven by a single-order parameter. (*b*) Volume strain, *V*_s_, data have been fit with standard solutions to a Landau expansion assuming that *V*_s_ scales with the square of the order parameter. On this basis the transition is first order in character at *x* = 0, 0.10, 0.18 and second order at *x* = 0.46. (*c*) Strain–strain relationships: tetragonal strains and volume strains vary linearly with each other at each composition and, within experimental uncertainty, extrapolate to the origin. Same symbols as for Fig. 6 ▸.

Values for the reference parameter, *V*_0_, were obtained by fitting a straight line to data for the unit-cell volume above the transition and extrapolating to lower temperatures. The resulting strains 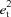 and *V*_s_ are reported in Figs. 8 ▸(*a*) and 8(*b*) as a function of *T*.

Symmetry rules determine the nature of coupling between the strain and order parameters. The order parameter *q*_JT_ and the tetragonal strain *e*_t_ transform as 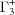 of 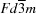, the active representation for the transition to *I*4_1_/*amd*, giving coupling of the form λ*e*_t_ *q*_JT_. *V*_s_, which does not break the cubic symmetry of the high-temperature phase, transforms as the identity representation and is proportional to 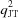. Therefore, the expected relationships between strain components are: 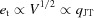. Two strains show a linear dependence when plotted as 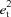 *versus* *V*_s_ (Fig. 8 ▸ *c*), consistent with these symmetry considerations.

The simultaneous linear and quadratic coupling of strain components to the Jahn–Teller order parameter implies a renormalization of the Landau expansion in order parameter for free energy (Landau & Lifshitz, 1958 ▸): the transition temperature is renormalized by coupling between the Jahn–Teller order parameter and the symmetry-breaking strain *e*_t_, while the fourth-order term of the expansion contains contributions from coupling of the square of *q*_JT_ with the volume strain.

With increasing Mg content, the magnitudes of the strains all decrease (Figs. 8 ▸ *a* and 8*b*). A decrease in the discontinuity at the transition and a trend towards linear behaviour are also fairly evident.

The 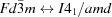 transition is required to be first order in character due to the existence of a third-order term in *q*_JT_. However, the strain variations with temperatures are not well represented by the standard solution for the order parameter
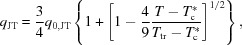
where *T*_tr_ is the transition temperature and 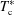 the renormalized critical temperature. Rather, the data for Cu100, Cu90 and Cu82 are well represented by the standard solution for a 246 solution with negative fourth-order coefficient (reported here, from Carpenter, Salje, Graeme-Barber, Wruck *et al.*, 1998 ▸)
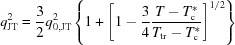
as shown in Figs. 8 ▸(*a*) and (*b*). At *T* = *T*_tr_, the jump in the value of *q*_JT_ from zero to *q*_0,JT_, is
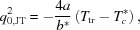
where *a* and b* here are the second- and the renormalized fourth-order terms in the Landau expansion, respectively.

**Figure 9:**
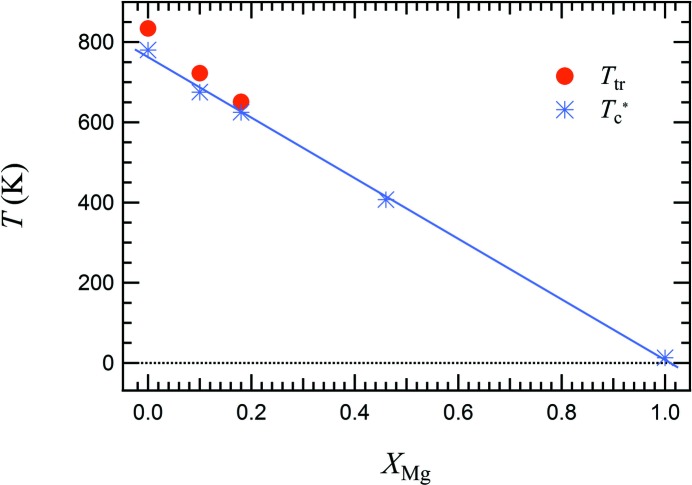
Variation of *T*_tr_ and 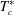 as a function of Mg content. Magnetic driven structural transition temperature (Nèel temperature) for MgCr_2_O_4_ (Kemei *et al.*, 2013 ▸) is also shown for comparison.

The equilibrium transition temperature *T*_tr_ for the three Cu-rich samples, as determined by 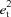 fits reported in Fig. 8 ▸, is 834 K (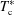 set to 780 K and 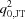 = 4.34 × 10^−3^) for Cu100, 722 K (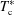 set to 675 K and 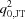 = 3.76 × 10^−3^) for Cu90, and 651 K (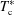 set to 625 K and 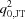 = 2.65 × 10^−3^) for Cu82. *T*_tr_ decreases linearly as a function of *X*_Mg_ as evident in Fig. 9 ▸, where the transition temperature for MgCr_2_O_4_ (data from Kemei *et al.*, 2013 ▸) is also shown for comparison. The difference between *T*_tr_ and 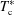, which is a measure of the extent of the first-order character of the transition, decreases systematically with increasing Mg content as well.

At *x* = 0.46, the transition conforms to 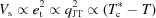, with 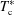 = 407 K, and thus appears to be second order in character.

This is all consistent with the third-order term being small for the Cu–Mg spinel system and the change from first-order to second-order character being due to changes in the value of the fourth-order coefficient. Coupling of the volume strain with the order parameter leads to a renormalization of the fourth-order coefficient and reductions in the strength of this coupling, as appears to occur with increasing Mg content, would contribute to this trend.

## 4. Summary   

Tetragonal distortion of Cu-rich members of the Mg*_x_*Cu_1 − *x*_Cr_2_O_4_ spinel solid solution, due to a cooperative Jahn–Teller effect, can be suppressed either by increasing temperature or by gradually replacing Cu^2+^ with Mg^2+^. The effect is to dilute the nearest-neighbour interactions of Cu^2+^ ions, thus reducing the efficiency of the cooperative distortion. With increasing the Mg content, a gradual reduction of the splitting of *a* and *c* unit-cell parameters and of the flattening of the tetrahedra is observed, and the transition temperature also decreases.

Jahn–Teller distortions of individual (Mg,Cu) tetrahedra are accompanied by variations in unit-cell parameters. Large spontaneous strains, coupling with an order parameter which originates physically from an electronic instability, lead to mean-field behaviour. The underlying role of strain in promoting long-range interactions is confirmed by the analysis of strain evolution, which shows that the Jahn–Teller phase transitions in CuCr_2_O_4_ conform to mean-field behaviour. Increasing Mg content causes reductions in the magnitude of the strains, and the two coupling coefficients also reduce with increasing Mg content. Reducing the coupling with the volume strain reduces in turn the renormalization of the fourth-term order Landau coefficient so that it goes from negative to positive, hence the tetragonal-to-cubic phase transition evolves from first order for Cu-rich samples towards second order at an intermediate composition.

## Supplementary Material

Crystal structure: contains datablock(s) Cu100_RT_CCD_T, Mg100_RT_CCD_C. DOI: 10.1107/S2052252516012574/lc5068sup1.cif

Structure factors: contains datablock(s) Cu100_RT_CCD_T. DOI: 10.1107/S2052252516012574/lc5068Cu100_RT_CCD_Tsup2.hkl

Structure factors: contains datablock(s) Mg100_RT_CCD_C. DOI: 10.1107/S2052252516012574/lc5068Mg100_RT_CCD_Csup3.hkl

Full crystal structure data. DOI: 10.1107/S2052252516012574/lc5068sup4.zip

CCDC references: 1497626, 1497627

## Figures and Tables

**Table 1:** Details of data collections and structure refinements of Mg*_x_*Cu_1 − *x*_Cr_2_O_4_ crystals at RT Standard deviations are in parentheses and refer to the last significant digits.

	Cu100	Cu90	Cu82	Cu57	Cu47	Mg100
Crystal size (mm)	0.5 × 0.5 × 0.5	0.2 × 0.15 × 0.15	0.2 × 0.2 × 0.2	0.15 × 0.1 × 0.1	0.1 × 0.1 × 0.1	0.15 × 0.15 × 0.1
Space group	*I*4_1_/*amd*	*I*4_1_/*amd*	*I*4_1_/*amd*	*I*4_1_/*amd*	*I*4_1_/*amd*	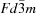
*a* (Å)	6.0287 (2)	6.0163 (1)	6.0045 (1)	5.9523 (1)	5.9435 (1)	8.3288 (1)
*c* (Å)	7.7803 (2)	7.8279 (1)	7.8778 (1)	8.0752 (2)	8.1379 (2)	–
Reflns measured	11 427	10 608	10 480	10 314	11 374	19 474
Reflns unique	4312	4375	4372	2400†	2700†	4570
Reflns independent	295	293	294	120	128	129
Twin fraction (%)	6.6 (3)‡	14.5 (2)	13.6 (2)	27.0 (3)	22.7 (2)	–
*R*_int_ (%)	2.40	2.04	2.41	1.65	1.93	1.78
Reflns with *I* > 2σ*_I_*	3682	3791	3822	2240	2582	4480
*R*_1_ (%)	2.50	2.03	2.28	4.38	2.54	1.71
*R*_all_ (%)	2.91	2.45	2.75	4.67	2.67	1.76
*wR*_2_ (%)	6.58	5.27	5.85	11.99	6.82	4.70
GOF	1.086	1.083	1.060	1.205	1.236	1.169
Δρ_max_ (e Å^−3^)	0.63	0.49	0.75	0.76	0.44	0.63
Δρ_min_ (e Å^−3^)	−1.14	−0.70	−1.03	−0.88	−0.59	−0.54

† Dataset limited to sin θ/λ = 0.7 Å^−1^.

‡ Three twin components with relative abundances of second and third components 3.7 (3), 2.9 (3).

**Table 2:** Tetragonal unit-cell parameters of Mg*_x_*Cu_1 − *x*_Cr_2_O_4_ crystals at different temperatures Standard deviations are in parentheses and refer to the last significant digits.

	Cu100		Cu90		Cu82			Cu54†	
*T* (K)	*a* (Å) × 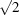	*c* (Å)	*a* (Å) × 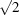	*c* (Å)	*a* (Å) × 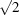	*c* (Å)	*T* (K)	*a* (Å) × 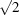	*c* (Å)
298	8.5249 (6)	7.7811 (10)	8.5103 (5)	7.8320 (11)	8.4924 (7)	7.8771 (10)	298	8.401 (3)	8.122 (4)
323	8.5264 (6)	7.7826 (10)	8.5128 (4)	7.8335 (6)	8.4914 (6)	7.8810 (8)	323	8.402 (2)	8.140 (2)
348	8.5257 (5)	7.7900 (8)	8.5124 (5)	7.8409 (5)	8.4900 (6)	7.8889 (8)	343	8.397 (2)	8.162 (3)
373	8.5251 (4)	7.7962 (7)	8.5118 (5)	7.8488 (7)	8.4913 (6)	7.8970 (7)	353	8.391 (4)	8.177 (6)
398	8.5252 (4)	7.8026 (7)	8.5117 (5)	7.8564 (5)	8.4899 (6)	7.9053 (7)	363	8.388 (2)	8.204 (4)
423	8.5253 (4)	7.8092 (8)	8.5105 (5)	7.8644 (7)	8.4890 (5)	7.9147 (5)	373	8.385 (3)	8.220 (4)
448	8.5257 (4)	7.8149 (8)	8.5102 (5)	7.8724 (7)	8.4880 (6)	7.9233 (6)	383	8.380 (5)	8.242 (9)
473	8.5249 (6)	7.8222 (9)	8.5094 (5)	7.8804 (5)	8.4849 (6)	7.9340 (8)	393	8.369 (5)	8.276 (9)
498	8.5253 (5)	7.8286 (10)	8.5081 (5)	7.8897 (6)	8.4843 (6)	7.9457 (6)	403	8.362 (4)	8.288 (7)
523	8.5239 (4)	7.8350 (7)	8.5089 (7)	7.8989 (9)	8.4826 (6)	7.9581 (5)	413	8.350 (3)	8.306 (6)
548	8.5237 (5)	7.8444 (9)	8.5058 (5)	7.9093 (6)	8.4789 (7)	7.9716 (7)	423	8.344 (2)	8.320 (3)
573	8.5229 (4)	7.8529 (7)	8.5046 (8)	7.9198 (9)	8.4750 (7)	7.9880 (6)	423‡	8.338 (3)	8.320 (6)
598	8.5224 (5)	7.8610 (8)	8.5016 (6)	7.9320 (8)	8.4699 (11)	8.0072 (15)	–	–	–
623	8.5215 (5)	7.8706 (8)	8.4981 (8)	7.9467 (9)	8.4590 (10)	8.0337 (15)	–	–	–
648	8.5188 (5)	7.8813 (8)	8.4940 (8)	7.9620 (11)	–	–	–	–	–
673	8.5182 (4)	7.8921 (7)	8.4892 (12)	7.9811 (20)	–	–	–	–	–
698	8.5155 (5)	7.9043 (8)	–	–	–	–	–	–	–
723	8.5129 (5)	7.9169 (9)	–	–	–	–	–	–	–
748	8.5095 (5)	7.9337 (9)	–	–	–	–	–	–	–
773	8.5055 (4)	7.9479 (8)	–	–	–	–	–	–	–
788	8.4998 (4)	7.9655 (7)	–	–	–	–	–	–	–
803	8.4955 (5)	7.9793 (8)	–	–	–	–	–	–	–
818	8.4899 (5)	7.9964 (7)	–	–	–	–	–	–	–

† Crystal size: 0.15 × 0.15 × 0.15 mm.

‡ On cooling.

**Table 3:** Cubic unit-cell parameters of Mg*_x_*Cu_1 − *x*_Cr_2_O_4_ crystals at different temperatures Standard deviations are in parentheses and refer to the last significant digits. Some intermediate data have been omitted in the table but are present in the graphs.

*T* (K)	Cu100	Cu90	Cu82	Cu54	Mg100
298	–	–	–	–	8.3312 (4)
323	–	–	–	–	8.3304 (4)
373	–	–	–	–	8.3342 (4)
423	–	–	–	–	8.3369 (3)
433	–	–	–	8.337 (4)	–
473	–	–	–	8.337 (2)	8.3391 (4)
523	–	–	–	8.339 (2)	8.3420 (5)
573	–	–	–	8.340 (2)	8.3458 (4)
623	–	–	–	8.347 (2)	8.3491 (5)
673	–	–	–	8.348 (2)	8.3523 (4)
698	–	–	8.3314 (5)	–	–
723	–	–	8.3333 (5)	8.353 (2)	8.3557 (5)
773	–	8.3393 (6)	8.3372 (5)	8.356 (2)	8.3571 (5)
823	–	8.3424 (6)	8.3420 (4)	8.358 (2)	8.3616 (5)
873	8.3411 (5)†	8.3448 (6)	8.3446 (4)	8.359 (2)	8.3635 (5)
898	8.3431 (4)	8.3466 (6)	–	–	–
923	8.3465 (7)	8.3492 (5)	8.3502 (5)	8.363 (2)	8.3686 (5)
973	8.3505 (9)	8.3523 (6)	8.3498 (5)	8.366 (2)	8.3711 (5)
1023	8.3546 (11)	8.3564 (6)	8.3545 (5)	8.369 (2)	8.3734 (5)
1073	8.3557 (7)	8.3601 (6)	8.3572 (4)	8.372 (2)	8.3769 (5)
1123	8.3609 (6)	–	8.3626 (5)	8.374 (2)	8.3797 (5)
1173	8.3622 (4)	8.3667 (6)	8.3665 (5)	8.378 (2)	8.3828 (6)

† Measured at 863 K.

**Table 4:** Details on data collections and structure refinements of Mg*_x_*Cu_1 − *x*_Cr_2_O_4_ crystals at HT

	Reflns measured	Reflns independent	*R*_int_ (%)	Reflns *I* > 2σ*_I_*	*R*_1_ (%)	*R*_all_ (%)	*wR*_2_ (%)	GOF	Δρ_max_ (e Å^−3^)	Δρ_min_ (e Å^−3^)
Cu100										
298 K	187	89	3.31	76	2.11	2.76	5.41	1.143	0.41	−0.77
373 K	187	89	3.73	77	2.71	3.12	5.71	1.111	0.59	−1.15
473 K	191	91	3.57	79	2.72	3.19	5.93	1.060	0.53	−1.21
573 K	191	91	3.04	78	2.72	3.40	7.01	1.158	0.56	−1.06
673 K	192	92	3.41	78	2.71	3.30	6.10	1.175	0.64	−1.03
773 K	195	93	2.74	78	2.61	3.49	6.21	1.112	0.45	−0.87
803 K	195	93	2.59	78	2.74	3.54	6.84	1.142	0.64	−1.00
863 K	192	45†	5.32	41	3.34	3.72	7.47	1.329	0.91	−0.53
973 K	192	45†	11.49	37	7.05	7.68	10.50	1.422	3.25	−1.01
1073 K	192	45†	19.08	38	8.42	9.26	12.44	1.190	3.70	−0.86
										
Cu90										
298 K	191	91	1.54	78	1.87	2.62	4.86	1.114	0.51	−0.69
373 K	191	91	2.25	75	2.23	3.16	5.84	1.171	0.60	−0.91
473 K	191	91	2.21	74	2.53	3.43	6.70	1.152	0.54	−0.92
573 K	193	92	2.73	74	2.42	3.45	6.15	1.127	0.50	−0.94
673 K	196	93	2.83	79	3.08	3.77	6.84	1.138	0.63	−1.16
773 K	192	45†	2.13	37	1.44	2.14	3.41	1.247	0.34	−0.33
873 K	192	45†	2.41	36	1.66	2.45	5.41	1.432	0.46	−0.48
973 K	192	45†	2.58	38	1.73	2.35	5.17	1.253	0.34	−0.42
1073 K	192	45†	2.55	38	1.67	2.65	5.04	1.230	0.43	−0.44
1173 K	192	45†	2.67	38	1.82	3.09	5.68	1.304	0.67	−0.61
										
Cu82										
298 K	191	91	2.20	76	3.59	4.16	9.15	1.219	0.65	−1.59
373 K	193	92	2.85	76	3.48	4.61	8.53	1.167	0.70	−1.64
473 K	387	93	3.02	76	3.22	4.36	8.39	1.064	0.89	−1.31
573 K	196	93	3.44	80	3.89	4.53	9.25	1.147	0.71	−1.47
623 K	388	93	3.90	76	3.50	4.36	8.64	1.186	0.82	−1.55
673 K	192	45†	14.20	37	6.65	7.96	12.92	1.090	3.03	−1.80
723 K	192	45†	2.02	37	1.41	2.11	5.24	1.248	0.39	−0.29
823 K	756	45†	2.16	38	1.68	2.43	4.90	1.199	0.34	−0.33
923 K	192	45†	2.14	38	1.59	2.40	4.88	1.312	0.26	−0.30
1023 K	192	45†	2.54	38	1.67	2.47	5.39	1.298	0.42	−0.30
										
Cu54										
298 K	196	93	6.50	78	4.56	5.49	10.00	1.115	0.85	−0.80
353 K	197	94	11.09	74	4.83	6.00	11.79	1.121	0.98	−0.75
473 K	728	45†	2.47	41	2.93	3.25	6.51	1.134	0.62	−0.62
673 K	192	45†	2.90	40	3.41	3.76	7.62	1.105	0.52	−0.79
873 K	192	45†	3.04	41	3.59	3.79	8.33	1.186	0.47	−0.83
1073 K	192	45†	2.97	40	3.35	3.89	7.26	1.210	0.61	−0.77
423 K‡	201	96	2.51	85	2.31	2.85	6.22	1.114	0.48	−0.47
										
Mg100										
298 K	192	45†	1.94	43	2.25	2.27	8.32	1.351	0.44	−0.48
523 K	192	45†	2.11	39	2.01	3.00	5.53	1.333	0.62	−0.41
973 K	756	45†	2.77	39	2.43	3.38	6.04	1.217	0.69	−0.48

† Cubic.

‡ On cooling

**Table 5:** Electron microprobe analyses and selected geometrical parameters for Mg*_x_*Cu_1 − *x*_Cr_2_O_4_ crystals at RT Standard deviations are in parentheses and refer to the last significant digits. OAV = Octahedral Angle Variance; OQE = Octahedral Quadratic Elongation; TAV = Tetrahedral Angle Variance; TQE = Tetrahedral Quadratic Elongation (Robinson *et al.*, 1971 ▸).

	Cu100	Cu90	Cu82	Cu57	Cu47	Mg100
*x* (site occupancy)	0	0.103 (1)	0.183 (1)	0.442 (5)	0.504 (2)	1
*x* (EPMA)†	0 (0)	0.103 (21)	0.178 (28)	0.428 (41)	0.527 (7)	–
*a* 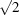 /*c*	1.0958 (3)	1.0869 (2)	1.0779 (2)	1.0424 (3)	1.0328 (3)	1
*V*_octahedron_ (Å^3^)	10.31	10.30	10.30	10.33	10.37	10.41
Cr—O_axial_ (Å) ×2	1.9854 (5)	1.9852 (4)	1.9865 (4)	1.9807 (13)	1.9872 (6)	1.9927 (1)
Cr—O_equatorial_ (Å) ×4	1.9884 (3)	1.9875 (2)	1.9870 (3)	1.9911 (9)	1.9915 (4)	1.9927 (1)
Cr—O_average_ (Å)	1.9874 (3)	1.9867 (2)	1.9869 (2)	1.9876 (7)	1.9990 (4)	1.9927 (1)
OAV (°)	37.26	36.99	36.56	34.11	34.10	33.35
OQE	1.0101	1.0100	1.0099	1.0091	1.0091	1.0088
Cr—Cr (Å)	2.8856 (1)	2.8904 (1)	2.8958 (1)	2.9162 (1)	2.9248 (1)	2.9447 (1)
*V*_tetrahedron_ (Å^3^)	3.71	3.74	3.77	3.85	3.87	3.90
(Cu,Mg)—O (Å)	1.9598 (4)	1.9607 (4)	1.9615 (4)	1.9616 (12)	1.9646 (6)	1.9660 (3)
O—(Cu,Mg)—O (°) ×2	103.40 (1)	103.89 (1)	104.39 (1)	106.80 (4)	107.29 (2)	109.47
O—(Cu,Mg)—O (°) ×4	122.45 (3)	121.31 (2)	120.21 (3)	114.97 (8)	113.92 (4)	109.47
O⋯O edge (Å) ×2	3.0760 (5)	3.0879 (4)	3.0994 (5)	3.150 (2)	3.1644 (6)	3.2106 (2)
O⋯O edge (Å) ×4	3.4355 (6)	3.4183 (5)	3.4009 (6)	3.308 (2)	3.2938 (8)	3.2106 (2)
TAV (°)	96.86	80.96	66.78	17.81	11.72	0
TQE	1.0270	1.0223	1.0182	1.0047	1.0031	1.0000

† EPMA for sample Cu54 gave *x* = 0.460 (71).

**Table 6:** Selected geometrical parameters for CuCr_2_O_4_ (Cu100) at HT Standard deviations are in parentheses and refer to the last significant digits. OAV = Octahedral Angle Variance; OQE = Octahedral Quadratic Elongation; TAV = Tetrahedral Angle Variance; TQE = Tetrahedral Quadratic Elongation (Robinson *et al.*, 1971 ▸).

	298 K	373 K	473 K	573 K	673 K	773 K	803 K	863 K	973 K	1073 K
*V*_oct_ (Å^3^)	10.30	10.30	10.33	10.38	10.39	10.33	10.38	10.38	10.25	10.26
Cr—O_axial_ (Å) ×2	1.985 (5)	1.983 (5)	1.991 (4)	1.992 (5)	1.991 (5)	1.986 (4)	1.997 (5)	1.992 (4)	1.988 (9)	1.986 (4)
Cr—O_eq._ (Å) ×4	1.988 (2)	1.989 (2)	1.988 (2)	1.992 (3)	1.993 (2)	1.990 (2)	1.990 (3)	1.992 (4)	1.985 (4)	1.986 (4)
Cr—O_av._ (Å)	1.987 (3)	1.987 (3)	1.989 (3)	1.992 (4)	1.992 (3)	1.989 (3)	1.992 (4)	1.992 (4)	1.985 (4)	1.986 (4)
OAV (°)	37.42	37.71	38.18	36.36	36.69	39.83	38.00	36.60	44.97	45.38
OQE	1.0102	1.0102	1.0103	1.0098	1.0099	1.0107	1.0102	1.0097	1.0118	1.0120
Cr—Cr (Å)	2.8855 (2)	2.8881 (1)	2.8925 (2)	2.8973 (1)	2.9031 (1)	2.9102 (2)	2.9138 (2)	2.9490 (2)	2.9523 (3)	2.9542 (2)
*V*_tetr._ (Å^3^)	3.71	3.74	3.75	3.76	3.80	3.88	3.87	3.96	4.08	4.09
Cu—O (Å)	1.960 (4)	1.962 (4)	1.965 (4)	1.963 (5)	1.967 (4)	1.977 (4)	1.974 (4)	1.976 (7)	1.996 (7)	1.998 (8)
O—Cu—O (°) ×2	103.4 (1)	103.6 (1)	103.6 (1)	103.9 (1)	104.3 (1)	105.0 (1)	105.0 (1)	109.47	109.47	109.47
O—Cu—O (°) ×4	122.4 (3)	122.0 (3)	121.9 (2)	121.2 (3)	120.3 (3)	118.9 (2)	118.8 (3)	109.47	109.47	109.47
O⋯O edge (Å) ×2	3.077	3.084	3.089	3.093	3.107	3.136	3.132	3.226	3.253	3.252
O⋯O edge (Å) ×4	3.435	3.432	3.436	3.422	3.413	3.406	3.397	3.226	3.253	3.252
TAV (°)	96.52	90.18	89.22	80.05	68.37	52.10	50.37	0	0	0
TQE	1.027	1.0250	1.0247	1.0221	1.0187	1.0141	1.0136	1.0000	1.0000	1.0000

**Table 7:** Selected geometrical parameters for Mg_0.10_Cu_0.90_Cr_2_O_4_ (Cu90) at HT Standard deviations are in parentheses and refer to the last significant digits. OAV = Octahedral Angle Variance; OQE = Octahedral Quadratic Elongation; TAV = Tetrahedral Angle Variance; TQE = Tetrahedral Quadratic Elongation (Robinson *et al.*, 1971 ▸).

	298 K	373 K	473 K	573 K	673 K	773 K	873 K	973 K	1073 K	1173 K
*V*_oct._ (Å^3^)	10.31	10.34	10.36	10.36	10.39	10.38	10.36	10.41	10.42	10.46
Cr—O_axial_ (Å) ×2	1.990 (4)	1.990 (6)	1.990 (7)	1.990 (6)	1.999 (6)	1.991 (2)	1.991 (3)	1.994 (3)	1.994 (3)	1.997 (3)
Cr—O_eq._ (Å) ×4	1.986 (2)	1.989 (2)	1.990 (3)	1.990 (3)	1.989 (3)	1.991 (2)	1.991 (3)	1.994 (3)	1.994 (3)	1.997 (3)
Cr—O_av._ (Å)	1.988	1.989	1.990	1.990	1.992	1.991 (2)	1.991 (3)	1.994 (3)	1.994 (3)	1.997 (3)
OAV (°)	37.33	36.51	36.72	37.53	37.17	36.68	38.43	37.42	38.50	37.48
OQE	1.0101	1.0099	1.0099	1.0101	1.0100	1.0097	1.0102	1.0099	1.0102	1.0099
Cr—Cr (Å)	2.8914 (2)	2.8946 (1)	2.8995 (1)	2.9053 (2)	2.9130 (4)	2.9484 (2)	2.9503 (2)	2.9530 (2)	2.9557 (2)	2.9581 (2)
*V*_tetr._ (Å^3^)	3.74	3.75	3.78	3.82	3.85	3.96	3.99	3.99	4.01	4.01
(Cu,Mg)—O (Å)	1.962 (3)	1.962 (4)	1.965 (5)	1.969 (5)	1.971 (4)	1.976 (5)	1.981 (6)	1.981 (6)	1.985 (6)	1.984 (7)
O—(Cu,Mg)—O (°) ×2	103.8 (1)	104.0 (1)	104.3 (2)	104.6 (2)	105.0 (1)	109.47	109.47	109.47	109.47	109.47
O—(Cu,Mg)—O (°) ×4	121.6 (2)	121.2 (3)	120.5 (4)	119.7 (3)	118.8 (3)	109.47	109.47	109.47	109.47	109.47
O⋯O edge (Å) ×2	3.087	3.091	3.102	3.117	3.128	3.227	3.235	3.234	3.241	3.240
O⋯O edge (Å) ×4	3.424	3.418	3.411	3.405	3.392	3.227	3.235	3.234	3.241	3.240
TAV (°)	84.57	78.94	70.12	60.42	50.54	0	0	0	0	0
TQE	1.0234	1.0217	1.0192	1.0164	1.0136	1.0000	1.0000	1.0000	1.0000	1.0000

**Table 8:** Selected geometrical parameters for Mg_0.18_Cu_0.82_Cr_2_O_4_ (Cu82) at HT Standard deviations are in parentheses and refer to the last significant digits. OAV = Octahedral Angle Variance; OQE = Octahedral Quadratic Elongation; TAV = Tetrahedral Angle Variance; TQE = Tetrahedral Quadratic Elongation (Robinson *et al.*, 1971 ▸).

	298 K	373 K	473 K	573 K	623 K	673 K	723 K	823 K	923 K	1023 K
*V*_oct._ (Å^3^)	10.29	10.29	10.37	10.24	10.36	10.44	10.43	10.41	10.43	10.43
Cr—O_axial_ (Å) ×2	1.983 (7)	1.989 (8)	1.997 (7)	1.984 (8)	1.986 (7)	1.995 (7)	1.994 (3)	1.993 (3)	1.995 (3)	1.995 (3)
Cr—O_eq._ (Å) ×4	1.987 (4)	1.985 (4)	1.988 (4)	1.984 (4)	1.993 (4)	1.995 (7)	1.994 (3)	1.993 (3)	1.995 (3)	1.995 (3)
Cr—O_av._ (Å)	1.986	1.986	1.991	1.984	1.990	1.995 (7)	1.994 (3)	1.993 (3)	1.995 (3)	1.995 (3)
OAV (°)	37.22	38.40	35.60	41.73	35.76	34.65	33.39	35.50	36.03	36.65
OQE	1.0100	1.0103	1.0096	1.0111	1.0095	1.0092	1.0089	1.0094	1.0095	1.0097
Cr—Cr (Å)	2.8958 (2)	2.8990 (2)	2.9041 (2)	2.9115 (2)	2.9165 (3)	2.950 (1)	2.9463 (2)	2.9493 (1)	2.9522 (2)	2.9538 (2)
*V*_tetr._ (Å^3^)	3.78	3.80	3.79	3.92	3.87	3.94	3.91	3.95	3.97	3.98
(Cu,Mg)—O (Å)	1.963 (6)	1.967 (6)	1.962 (6)	1.980 (6)	1.968 (6)	1.973 (13)	1.967 (6)	1.974 (5)	1.977 (6)	1.979 (6)
O—(Cu,Mg)—O (°) ×2	104.5 (2)	104.5 (2)	104.7 (2)	105.4 (2)	106.1 (2)	109.47	109.47	109.47	109.47	109.47
O—(Cu,Mg)—O (°) ×4	120.1 (4)	120.0 (4)	119.6 (4)	117.9 (4)	116.5 (4)	109.47	109.47	109.47	109.47	109.47
O⋯O edge (Å) ×2	3.104	3.110	3.107	3.151	3.145	3.221	3.212	3.224	3.229	3.233
O⋯O edge (Å) ×4	3.402	3.408	3.392	3.391	3.348	3.221	3.212	3.224	3.229	3.233
TAV (°)	64.95	64.79	59.14	41.07	29.37	0	0	0	0	0
TQE	1.0177	1.0177	1.0161	1.0110	1.0078	1.0000	1.0000	1.0000	1.0000	1.0000

**Table 9:** Selected geometrical parameters for Mg_0.46_Cu_0.54_Cr_2_O_4_ [Cu54, *x* (EPMA) = 0.460(71)] at HT Standard deviations are in parentheses and refer to the last significant digits. OAV = Octahedral Angle Variance; OQE = Octahedral Quadratic Elongation; TAV = Tetrahedral Angle Variance; TQE = Tetrahedral Quadratic Elongation (Robinson *et al.*, 1971 ▸).

	298 K	353 K	473 K	673 K	873 K	1073 K	423 K†
*V*_oct._ (Å^3^)	10.23	10.32	10.38	10.47	10.45	10.59	10.45
Cr—O_axial_ (Å) ×2	1.978 (7)	1.982 (10)	1.992 (3)	1.997 (3)	1.996 (3)	2.004 (3)	1.989 (5)
Cr—O_eq._ (Å) ×4	1.985 (5)	1.990 (6)	1.992 (3)	1.997 (3)	1.996 (3)	2.004 (3)	1.998 (3)
Cr—O_av._ (Å)	1.983	1.987	1.992 (3)	1.997 (3)	1.996 (3)	2.004 (3)	1.995
OAV (°)	38.76	36.58	36.04	33.79	36.93	32.74	32.00
OQE	1.0103	1.0097	1.0095	1.0090	1.0098	1.0087	1.0085
Cr—Cr (Å)	2.9212 (9)	2.929 (1)	2.9478 (8)	2.9514 (5)	2.9555 (6)	2.9600 (6)	2.945 (1)
*V*_tetr._ (Å^3^)	3.92	3.92	3.95	3.93	3.99	3.95	3.89
(Cu,Mg)—O (Å)	1.973 (7)	1.971 (9)	1.974 (5)	1.972 (6)	1.981 (6)	1.975 (6)	1.964 (4)
O—(Cu,Mg)—O (°) ×2	107.3 (2)	107.8 (3)	109.47	109.47	109.47	109.47	109.5 (1)
O—(Cu,Mg)—O (°) ×4	114.0 (4)	112.8 (5)	109.47	109.47	109.47	109.47	109.4 (3)
O⋯O edge (Å) ×2	3.177	3.186	3.224	3.220	3.235	3.225	3.205
O⋯O edge (Å) ×4	3.308	3.284	3.224	3.220	3.235	3.225	3.208
TAV (°)	11.86	6.60	0	0	0	0	0.01
TQE	1.0031	1.0017	1.0000	1.0000	1.0000	1.0000	1.0000

† On cooling.

**Table 10:** Selected geometrical parameters for MgCr_2_O_4_ (Mg100) at HT Standard deviations are in parentheses and refer to the last significant digits.

	298 K	523 K	973 K
*V*_oct._ (Å^3^)	10.41	10.47	10.52
Cr—O (Å)	1.993 (3)	1.996 (3)	2.000 (3)
OAV (°)	33.93	33.10	35.71
OQE	1.0090	1.0088	1.0095
Cr—Cr (Å)	2.9455 (1)	2.9493 (2)	2.9596 (2)
*V*_tetr._ (Å^3^)	3.91	3.92	3.99
Mg—O (Å)	1.968 (6)	1.969 (5)	1.981 (6)
O⋯O edge (Å)	3.214	3.215	3.236
